# Layered Ferroelectric NbOI_2_ Flakes Toward In‐Plane Anisotropic Self‐Powered Sensing

**DOI:** 10.1002/smsc.202300125

**Published:** 2023-11-29

**Authors:** Xuzhou Sun, Yingjie Wan, Yuqiang Fang, Fuqiang Huang

**Affiliations:** ^1^ State Key Laboratory of High Performance Ceramics and Superfine Microstructure Shanghai Institute of Ceramics Chinese Academy of Sciences Shanghai 200050 China; ^2^ Center of Materials Science and Optoelectronics Engineering University of Chinese Academy of Sciences Beijing 100049 China; ^3^ Beijing National Laboratory for Molecular Sciences and State Key Laboratory of Rare Earth Materials Chemistry and Applications College of Chemistry and Molecular Engineering Peking University Beijing 100871 China; ^4^ School of Materials Science and Engineering Shanghai Jiao Tong University 800 Dongchuan Road Shanghai 200240 China

**Keywords:** in-plane anisotropic sensing, piezoelectric nanogenerators, 2D NbOI_2_ flake

## Abstract

2D ferroelectric materials have attracted much interest due to their potential for developing flexible self‐powered nanogenerators. Niobium oxide diiodide (NbOI_2_) has in‐plane anisotropy of electrical properties and large lateral piezoelectric coefficient, which makes it possess high‐performance and unique behavior in flexible sensing. In this work, multidirectional piezoelectric nanogenerator (PENG) devices using NbOI_2_ flake are fabricated and excellent energy harvesting and sensing capabilities are found. Specifically, the NbOI_2_‐based PENG can exhibit a long‐time stable voltage output of 215 mV at a large strain of 1.1%. More importantly, the periodic output signal pattern of the twelve‐electrode NbOI_2_‐based PENG in six directions is investigated, and this anisotropy provides the possibility of achieving simultaneous signal harvesting in multiple directions. This work broadens the scope of applications of 2D materials in nano‐energy and provides new ideas and insights for further exploration of nano‐energy and smart wearable nano‐electronic devices.

## Graphical abstract

Layered NbOI_2_ flakes with large piezoelectric coefficient are prepared into piezoelectric nanogenerator devices for energy harvesting and sensing capabilities. A high voltage response of 215 mV and anisotropic electrical signals can be achieved. In this work, new ideas and insights for further exploration of nano‐energy and smart wearable devices are provided.

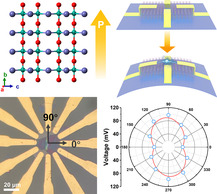

## 1 Introduction

Flexible and wearable bioelectronics are influencing and shaping the way people communicate with their surroundings.^[^ ^1^, ^2^, ^3^, ^4^ ^]^ To achieve the need for easily deformable, attachable, and lightweight biosensing, there is an urgent need to develop flexible sensing devices that are distributed and do not rely on external power sources.^[^ ^5^, ^6^ ^]^ Piezoelectric nanogenerators (PENGs) can convert small mechanical irritations of the environment into electrical signals for energy harvesting,^[^ ^7^, ^8^, ^9^ ^]^ showing potential applications in self‐powered systems,^[^ ^10^, ^11^, ^12^ ^]^ sensing devices,^[^ ^13^, ^14^, ^15^ ^]^ and wearable electronics.^[^ ^16^, ^17^, ^18^ ^]^ For the fabrication of PENG, conventional piezoelectric ceramic films are limited due to their brittleness.^[^ ^19^, ^20^ ^]^ In contrast, 2D piezoelectric materials are promising candidates for highly sensitive and flexible PENG devices due to their lightweight, planar integrity, and ability to withstand large strains.^[^ ^21^, ^22^, ^23^, ^24^ ^]^

Recently, 2D piezoelectric materials including SnS, SnSe, MoS_2_, and CuInP_2_S_6_ have been successfully applied to prepare PENGs.^[^ ^25^, ^26^, ^27^, ^28^ ^]^However, most 2D crystals do not exhibit strong intrinsic piezoelectric effects so far and often require complex atomic‐level engineering methods to induce or enhance the piezoelectric properties, which increases the difficulty of moving them toward practical applications.^[^ ^29^, ^30^, ^31^, ^32^ ^]^ In addition, for 2D piezoelectric materials with in‐plane anisotropy, electrical signal collection is always limited by the orientation of the measurement electrodes and for materials with multiangle signals cannot be collected. More importantly, the output voltage available for 2D piezoelectric materials up to now is not satisfactory to show the prospect of future industrial conversion.

Recently, a class of layered transition metal oxyhalides NbOX_2_ (X = Cl, Br, I) has received much attention because of their in‐plane anisotropic ferroelectric and optical properties.^[^ ^33^, ^34^, ^35^, ^36^ ^]^ One of these materials, NbOI_2_, has been determined to have great intrinsic piezoelectricity, and the piezoelectric effect is independent of the crystal thickness.^[^ ^37^ ^]^ Specifically, Wu et al. measured that the lateral piezoelectric coefficient (*d*_11_) of NbOI_2_ is ≈21.8 pm V^−1^, which was significantly higher than the concurrent measurements of α‐In_2_Se_3_ and CuInP_2_S_6_. It is foreseen that if this material is prepared as a PENG, it will provide a powerful output voltage. Since the directions for the inversion‐symmetry‐breaking distortions are the same in all layers, the piezoelectric properties of NbOI_2_ in monolayer and thin flake form are similar. This means that the processes required to prepare the material into devices can be substantially simplified. In addition, the strong anisotropy of NbOI_2_ offers the possibility of measuring multiangle signals. Therefore, the combination of NbOI_2_‐layered materials into flexible and wearable PENGs will have great potential for applications.

Here, we obtained large‐area NbOI_2_ flakes by mechanical exfoliation method. Several NbOI_2_‐based PENGs device containing up to multiple electrodes were prepared by photolithography, and the output signals of NbOI_2_ at multiple angles in the in‐plane were investigated for the first time. The NbOI_2_‐based PENG exhibited stable voltage signal responses of 215 and 30 mV in the directions of the optimal and worst signals, respectively, with the two directions perpendicular to each other. Further, we measured up to six directions spaced 30° apart on the same NbOI_2_ film, and this anisotropic piezoelectric signal response was symmetrically distributed with angle. This signal variability can be attributed to the relationship between O–Nb–O chains and I–Nb–I chains presenting ≈90° angles within the NbOI_2_ face. In addition, the NbOI_2_‐based PENGs can be attached to the human body, demonstrating excellent biosensing performance and enabling more accurate recognition of organisms in motion and speech.

## 2 Results and Discussion

### 2.1 NbOI_2_ Characterization

**Figure 1:**
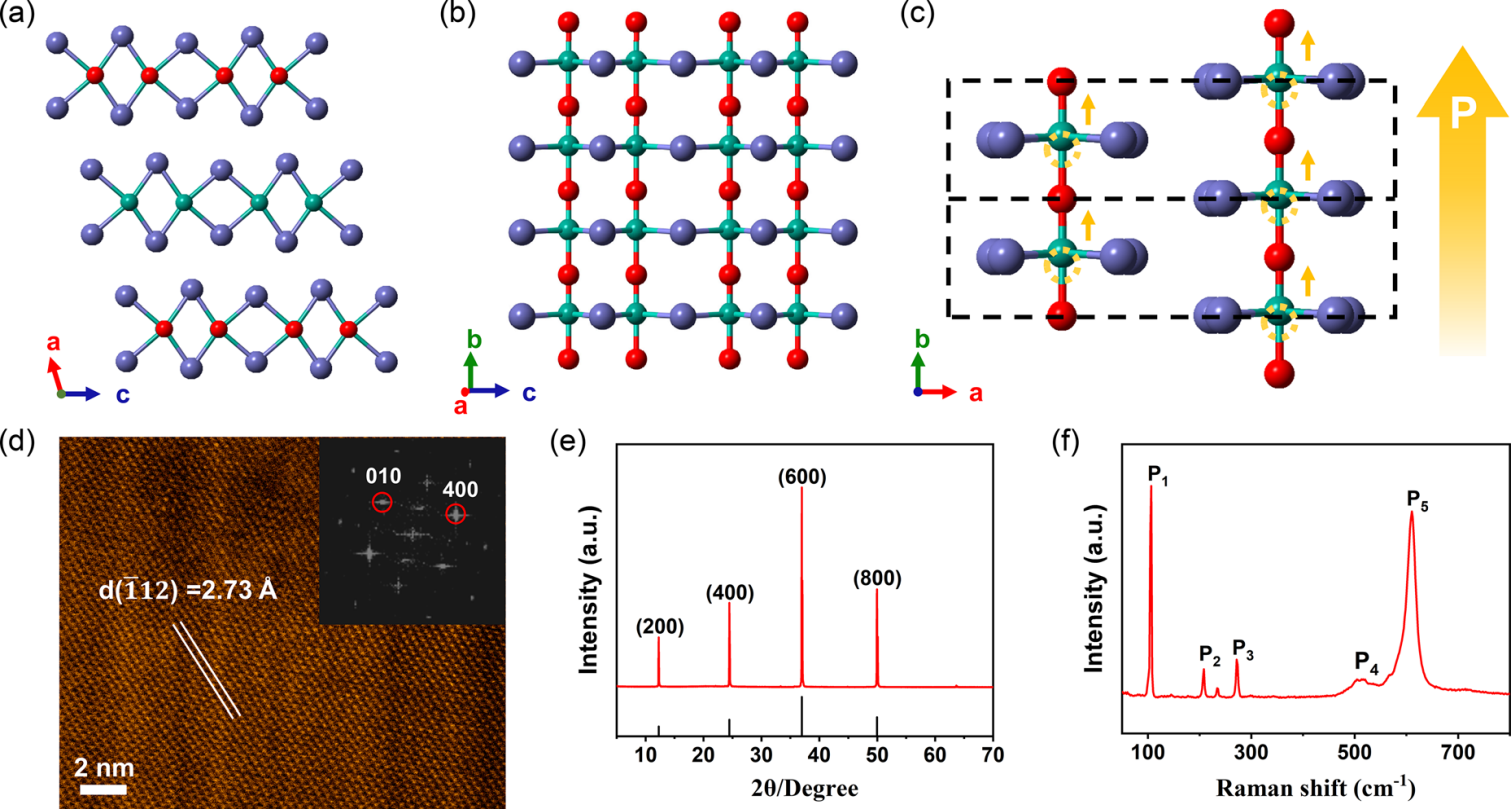
Characterization of NbOI_2_. a) Side view along the *b*‐axis and b) top view to the crystal structure of NbOI_2_. Green balls denote Nb atoms; purple balls denote I atoms; red balls denote O atoms. c) Schematic, viewed along the *c*‐axis, shows that Nb atoms are far from the equilibrium position along the *b*‐axis. The yellow circles denote the position of the equilibrium center of the [NbO_2_I_4_] octahedron; the arrows denote the direction of spontaneous polarization. d) STEM image of the 2D NbOI_2_ crystal. e) Powder X‐ray pattern and f) Raman spectrum of the NbOI_2_ crystals.

**Figure** **1**a,b shows the crystal structure of NbOI_2_, which consists of distorted [NbO_2_I_4_] octahedra connected by edge sharing of I–I edges along the *c*‐axis and by corner sharing of O atoms along the *b*‐axis. NbOI_2_ layer has a rectangular network‐like lattice, made of O–Nb–O and I–Nb–I chains with a near‐perpendicular relationship. Due to the pseudo‐Jahn–Teller effect, the Nb atoms are severely deviated from the octahedral center position, resulting in a spontaneous polarization along the *b*‐axis direction as shown in Figure 1c.^[^ ^37^ ^]^ We prepare many NbOI_2_ samples by mechanical exfoliation and transfer them to flexible PET substrates for subsequent PENG preparation. Contact mode AFM measurements indicate that the thickness of a mechanically exfoliated NbOI_2_ flake is about 70 nm (Figure S1, Supporting Information). Figure 1d shows an STEM of the exfoliated NbOI_2_ sample with a rectangular network‐like arrangement that corresponds to the lattice of Figure 1b. Figure 1e shows an X‐ray diffraction (XRD) pattern of the exfoliated NbOI_2_ flake, where four diffraction peaks reveal the selective orientation of the flake as (100). Figure 1f shows the Raman spectrum of NbOI_2_ with the presence of five peaks in the detection range (P_1_–P_5_ from low to high frequencies). It is worth mentioning that the exfoliated NbOI_2_ flakes always show a rectangular or elongated shape with nearly vertical edges, which is consistent with the STEM and the growth orientation. This rectangular morphology helps us to determine the lattice orientation directly from the optical microscopy images.

### 2.2 Piezoelectricity of NbOI_2_‐Based PENG Under Multiple Directions

**Figure 2:**
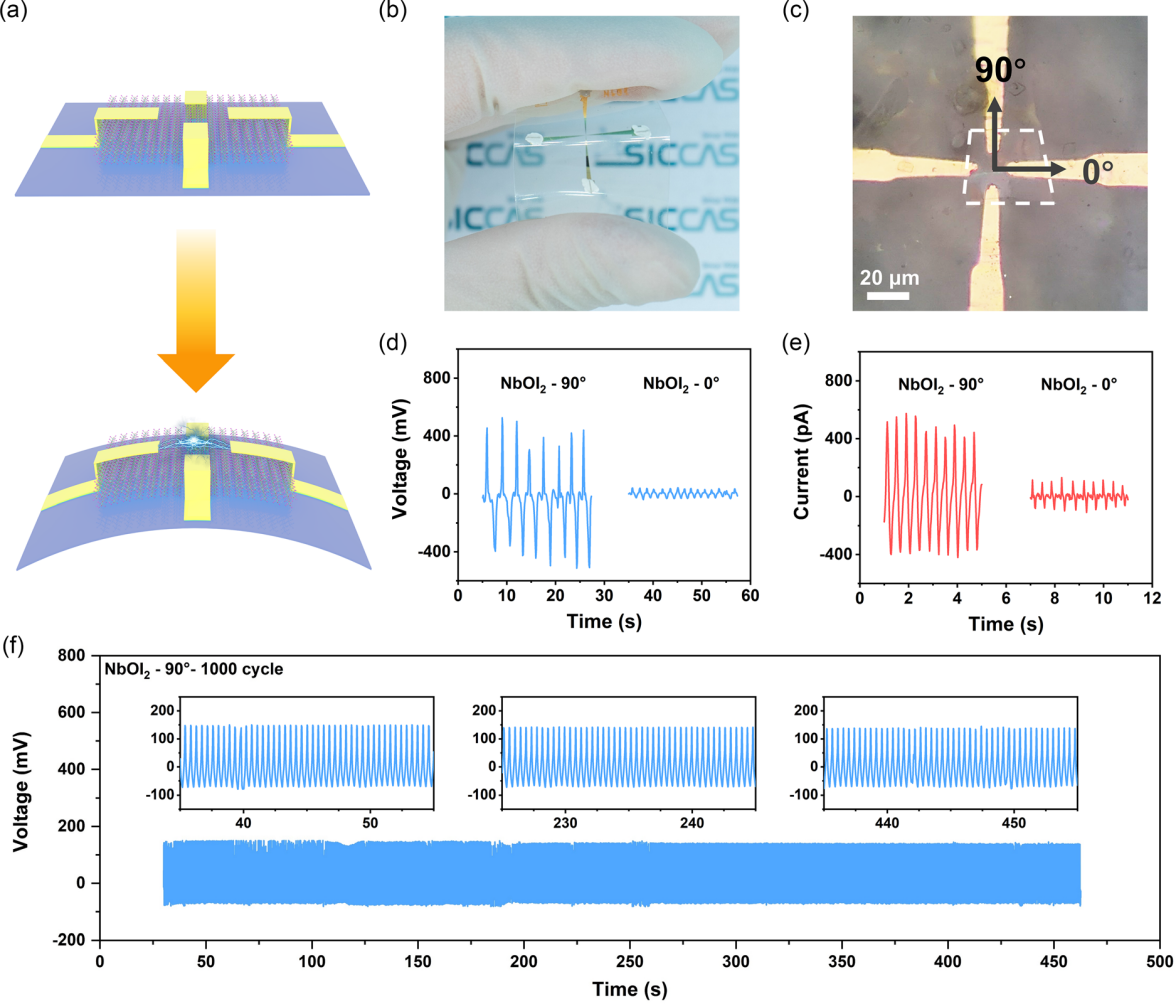
Performance of the four‐electrode NbOI_2_‐based PENG. a) Bending schematic of PENG. b) Photograph and c) optical microscopy image of the four‐electrode NbOI_2_‐based PENG. d) Open‐circuit voltage (*V*_OC_) and e) short‐circuit current (*I*_SC_) in two perpendicular directions. f) 1000 cycles of *V*_OC_ under 1.1% strain in 90° direction.

To initially evaluate the electrical performance of the device, a four‐electrode NbOI_2_‐based PENG was prepared as shown in **Figure** **2**b. The thickness of NbOI_2_ is about 70 nm (Figure S1, Supporting Information). Figure 2a shows the bending schematic of the four‐electrodes PENG. When a tensile strain is applied, a piezoelectric polarization charge with opposite polarity is generated at two ends of the NbOI_2_ sample to form an electrical potential difference. Once the squeezing force is released, the strain disappears and the piezoelectric potential on the electrodes decreases at the same time. Afterward, the accumulated charges on the electrodes move in the opposite direction, resulting in an electrical signal opposite to the previous process.

Figure 2c,d shows the detected *I*_SC_ and *V*_OC_ signals for the four‐electrodes device in both 90° and 0° directions, and the experiments were performed with one tensile strain and one compressive strain. These periodically varying signals also show that the 90° direction is a good signal direction in the four‐electrodes device: its *V*_OC_ and *I*_SC_ signals reach 400 mV and 450 pA. Respectively, the *V*_OC_ and *I*_SC_ signals in the 0° direction are only 60 mV and 85 pA, which are significantly weaker than those in the 90° direction. We did not observe significant voltage outputs on the bare PET substrate without transferring NbOI_2_, establishing that these signals were indeed from NbOI_2_ (Figure S2a, Supporting Information). Meanwhile, we performed unidirectional bending and stretching tests on the device to further check whether the electrical signals were generated by piezoelectric phenomena. When tensile strain was performed, a negative voltage signal was detected first, and a positive voltage signal was generated during rebound (Figure S2b, Supporting Information). In contrast, the output signals detected in compressive strain have the opposite shape (Figure S2c, Supporting Information). These output signals are reversible, proving that the variability of the electrical signals in the vertical direction indeed comes from the anisotropic piezoelectric effect of the NbOI_2_ material and not from the interference of external signals.

To demonstrate the durability of the NbOI_2_‐based PENG, and to further illustrate the difference in the same signals of the PENG in both directions, tensile cycling tests were performed at 1.1% of the same high strain in both directions. The method of strain calculation is described in the detail in Figure S3 (Supporting Information). As Figure 2g and Figure S4, Supporting Information show, after 1000 cycles, there are stable and continuous *V*_OC_ signals in both vertical directions. The signals in the 90° and 0° directions are stable at 215 and 30 mV. The *I*_SC_ at 180 pA is also stable for 1000 cycles under the same conditions (Figure S5, Supporting Information). In **Table** **1**, we compare the performance of NbOI_2_‐based PENG with other reported 2D PENG, and multilayer NbOI_2_ shows very good voltage signals.

**Table 1:** Performance of the NbOI_2_‐based PENG in this work compared with other 2D PENGs reported

	Voltage [mV]	Current [pA]	Thickness [nm]	Strain [%]	Piezoelectric response [pm V^−1^]	Refs.
Multilayer MoS_2_	15	20	0.6	0.64	2.91^[^ ^23^ ^]^	[27]
S‐treated MoS_2_	22	100	–	0.48	3.73	[38]
Multilayer MoSe_2_	55	–	0.8	0.6	3.05^[^ ^23^ ^]^	[39]
Bilayer WSe_2_	85	100	1.5	0.95	1.5	[21]
h‐BN nanoflakes	50	30	25	0.28	1.5^[^ ^23^ ^]^	[40]
Multilayer CuInP_2_S_6_	40	250	90	0.85	10	[41]
Multilayer SnS	300	160	0.7	0.7	5.95	[25]
SnS_2_ nanosheets	33	180	5	0.6	5	[42]
Multilayer SnSe	100	80	190	0.6	23	[26]
Multilayer NbOI_2_	215	180	70	1.1	21.8^[^ ^37^ ^]^	This work

**Figure 3:**
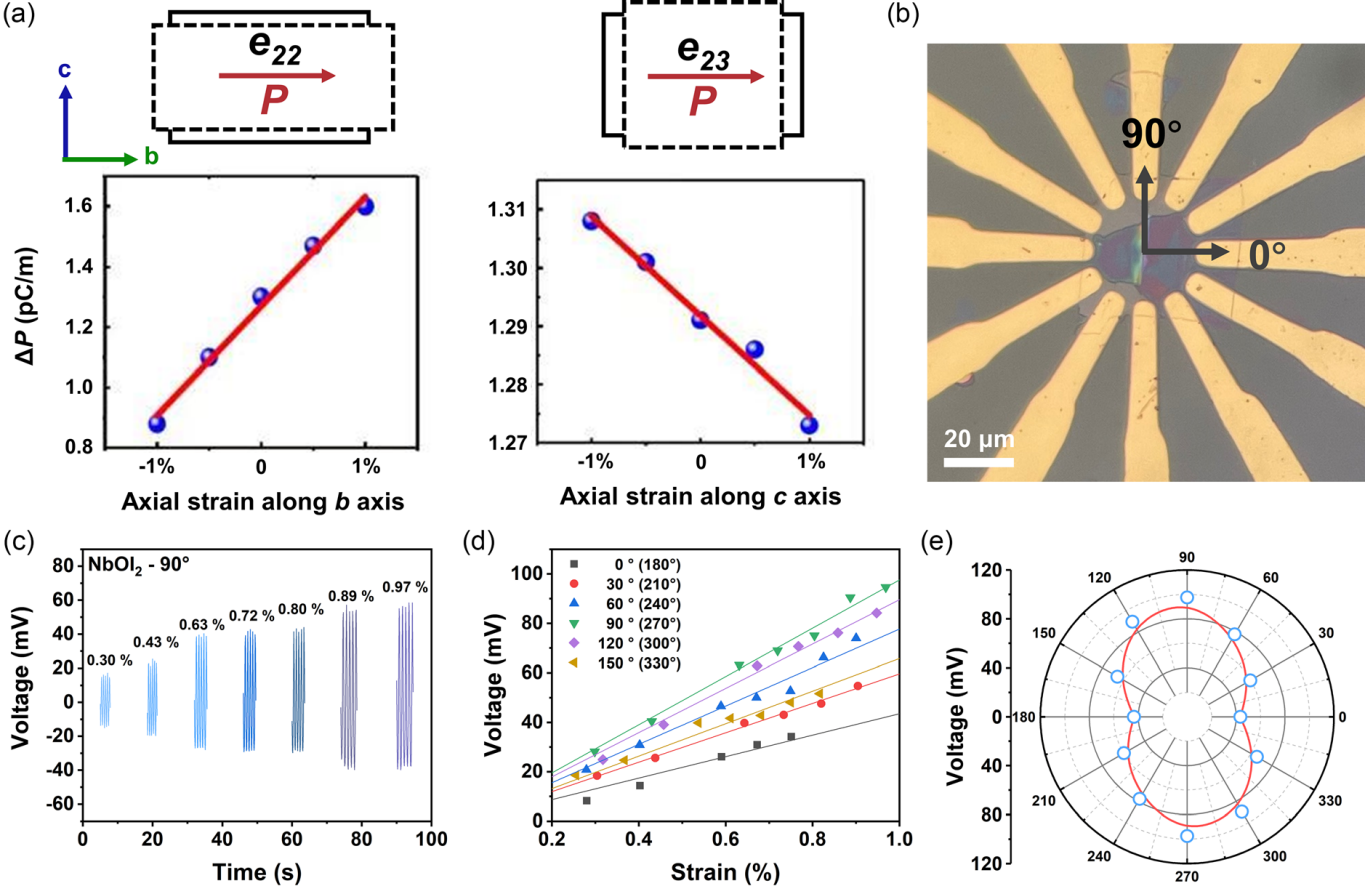
Performance of twelve‐electrode NbOI_2_‐based PENG. a) Theoretical calculation of polarization strength changes when stress is applied to polarized or unpolarized axes. b) Optical microscopy images of the twelve‐electrode NbOI_2_‐based PENG. c) Relationship between strain and output voltage at 90 degrees direction. d) Strain‐output voltage image and its linear fit at different directions. e) Polar coordinate images of output voltage in each direction at 1% strain after fitting.

It is exciting to exhibit anisotropic piezoelectric signal differences on the same sample, which represents the possibility of collecting information in multiple directions simultaneously using only one PENG. To illustrate the signal difference of NbOI_2_‐based PENG in the vertical direction, we simulate the polarization intensity when the NbOI_2_ crystal is stressed in different directions (**Figure** **3**a). Where the *b*‐axis direction is the in‐plane polarization axis direction where the O–Nb–O chain is located, and the vertical *c*‐axis direction is the nonpolarization axis direction where the I–Nb–I chain is located (Figure 1b). The change in polarization intensity ΔP = 0.7 pC m^−1^ when a 2% strain occurs in the axial direction along the *b*‐axis, while the axial strain along the *c*‐axis in the same case leads to a change of only ≈0.045 pC m^−1^. Although in NbOI_2_ crystals the *c*‐axis direction has a smaller resistance than the *b*‐axis direction due to the weaker electronegativity of the I atom, *R*_b_/*R*_c_ = 1.7.^[^ ^36^ ^]^ The difference in electrical signal due to the resistance is much smaller than the piezoelectric signal due to the polarization. The results show that the strongest direction of the electrical signal is always along the *b*‐axis.

To expand the range of orientations that can be detected by the NbOI_2_‐based PENG and to check the simulation results, we selected NbOI_2_ crystals with stripped dimensions larger than 40 um and fabricated a PENG with twelve equally spaced electrodes on it. Figure 3b shows an optical microscopy image of a twelve‐electrode NbOI_2_‐based PENG with a 30° separation maintained between each adjacent electrode. To ensure comparability of the multiangle signal, the tips of the twelve electrodes were controlled to all be on the same circle with a radius of 34 μm. Pairs of electrodes were connected for each test and bent in the direction perpendicular to the electrodes. Figure 3c shows the open‐circuit voltages at multiple tensile strains in a typical 90° direction, and at a single direction, the open‐circuit voltage increases with increasing strain, which is consistent with previous experimental observations.^[^ ^36^ ^]^ The *V*_OC_ in this direction is 94 mV at 0.97% strain. We further examine the *V*_OC_ in the remaining five directions for multiple tensile strains, and the relevant data are summarized in Figure 3d. To focus on comparing the differences in signals at different angles, we use linear fit over the zero point for *V*_OC_ and strain. The slope of the fit can represent the *V*_OC_ signal due to tensile strain in that direction at 1% strain. The piezoelectric signals at different angles are comparable only if the same strain is applied.

The slope information with angle is presented in Figure 3e. For the twelve‐electrode NbOI_2_‐based PENG, the *V*_OC_ in the 0°, 30°, 60°, 90°, 120°, and 150° directions are 43, 59, 78, 97, 89, and 66 mV. Because the electrodes are measured in pairs, the curves exhibit centrosymmetry, and the images show a distribution pattern similar to the number “8”. In this twelve‐electrode NbOI_2_‐based PENG, the 90° and 0° directions are the directions of the strongest and weakest piezoelectric signals, and both of their electrode directions are also parallel to the edges of the rectangular NbOI_2_ sheet,corresponding to the *b*‐axis and *c*‐axis in the lattice. The signals of the remaining four angles are approximately symmetrically distributed along the vertical line.

### 2.3 Energy Harvesting from the Human Body

**Figure 4:**
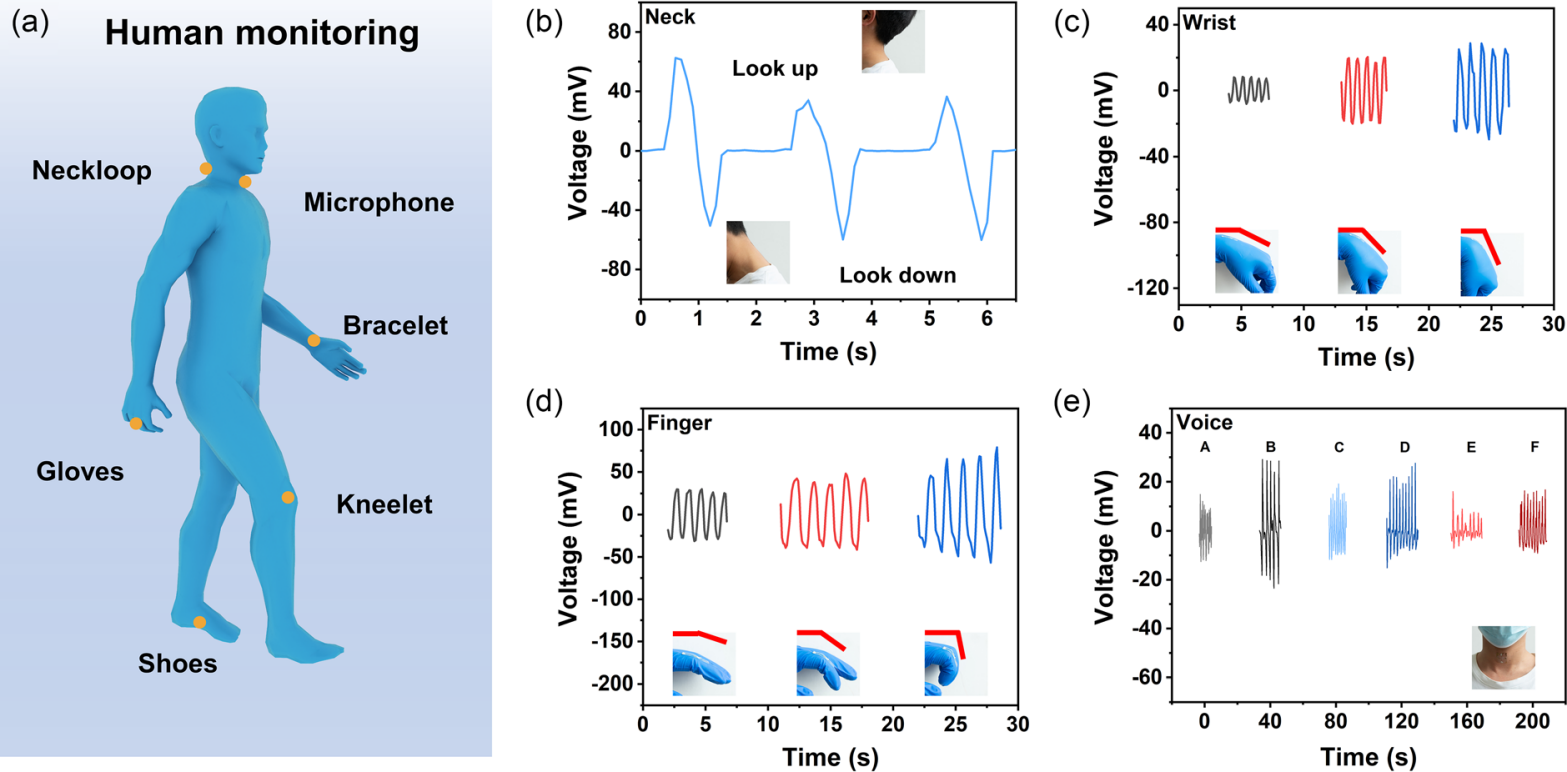
Application of NbOI_2_‐based PENG as a wearable device. a) NbOI_2_‐based PENGs can be used for a variety of human monitoring. b) The neck‐mounted device can recognize postural changes and respond to conditioning signals. c,d) Devices that collect energy from the wrist (c) and fingers (d) can recognize the degree of flexion. e) Devices attached to the throat can respond differentially to different letter sounds. No formal ethical approval was required for the experiments demonstrated herein. Written informed consent was obtained from all the participants prior to the publication of this study.

We explored the energy collection and flexible sensing of NbOI_2_‐based PENGs on the human body. Due to their small size and flexibility, the PENGs can be made into a variety of monitoring devices that are attached to the human body for sensing and collecting energy (**Figure** **4**a). No formal ethical approval was required for the experiments demonstrated herein. Written informed consent was obtained from all the participants prior to the publication of this study. Figure 4b shows the energy harvesting and motion feedback detection when the NbOI_2_‐based PENG is attached to the back of the neck. A voltage response of ≈40 mV can be generated when the tester tilts his head and lowers his head, and the electrical signal is directional, allowing discrimination between opposing forms of motion. In addition, as shown in Figure 4c,d, the PENG can respond to the bending curvature of the wrist and fingers, with the output voltage increasing with the bending angle. We then tried to attach the device to the throat to detect the vibrations caused by the vocal cords during speech. As shown in Figure 4e, the same tester pronounced “A”, “B”, “C”, “D”, “E”, and “F”, The PENG of NbOI_2_ effectively collects the vibration energy and converts it into a voltage signal. When pronouncing different letters, different voltage waveforms are obtained, and when pronouncing the same letter, the waveforms have similarity (Figure S6, Supporting Information). Based on the above tests, these NbOI_2_‐based PENGs can effectively collect the mechanical energy generated by human joints and convert it into electrical signals. Therefore, NbOI_2_‐based PENGs have great potential as wearable devices and self‐powered sensors in biomechanical energy collection and human activity monitoring.

## 3 Conclusions

We have successfully prepared a variety of NbOI_2_‐based PENGs with the help of photolithography. The NbOI_2_‐based PENGs have excellent stability, generating a stable signal output of about 215 mV and 180 pA for a long time at a large strain of 1.1%. In contrast, only 30 mV of voltage signal is available in the vertical direction for the same test conditions. Through theoretical simulations, we clarify that the signal difference in these two directions corresponds to the polarization difference between the mutually perpendicular O–Nb–O and I–Nb–I chain directions in the NbOI_2_ lattice. Combined with measurements of the PENG of the twelve‐electrode NbOI_2_ in six directions, it is verified that the electrical signals in the remaining directions are the sum of the components generated by the electrical signals in the directions of these two crystallographic axes. This anisotropy provides the possibility to achieve simultaneous signal harvesting in multiple directions. The NbOI_2_‐based PENGs can not only collect biomechanical energy from each joint on the human body but also effectively collect acoustic signals. This study broadens the scope of applications of 2D materials in the nano‐energy and provides new ideas and insights for further exploration of nano‐energy and smart wearable nano‐electronic devices.

## 4 Experimental Section

4.1

4.1.1

##### Material Synthesis

NbOI_2_ samples were synthesized by the chemical vapor transport reaction using stoichiometric elemental precursors of high‐purity Nb, Nb_2_O_5,_ and I_2_ with additional small amount of iodine as the transport agent. The precursors were sealed inside a quartz tube under high vacuum and were subjected to a two‐zone horizontal tube furnace for a reaction time of 7 days under 500–600 °C. The shiny rectangle crystals were collected from the cold end of the tube.

##### Device Fabrication

2D NbOI_2_ flakes were mechanically exfoliated by using Scotch tape from bulk NbOI_2_ crystals and then transferred onto an oxygen plasma dry etching pretreated flexible poly(ethylene terephthalate) (PET) substrate. The PET substrate was heated to 100 °C for 10 min during the transferring process to enhance adhesion force between it and NbOI_2_ flakes. A suitable size of NbOI_2_ flake is found on the substrate by the photolithography machine, and the expected electrode structure is drawn and photoengraved. Sequentially, Cr (10 nm) and Au (70 nm) thin layers were deposited on the PET substrate by sputtering, followed by a metal lift‐oﬀ process.

##### Material Characterization

An optical microscope (Moticam Pro 205A) equipped was used for optical imaging. The XRD characterization was carried out on a Bruker D8 Advance diffractometer operating with a Cu Kα radiation (λ = 1.5406 Å). Raman spectroscopy was conducted on a Jobin‐Yvon LabRAM HR‐800 spectrometer with a laser excitation wavelength of 532 nm. The thicknesses were accurately determined by using a contact mode AFM (AIST‐NT). STEM operating at 300 kV was used to examine crystal structures, of which the data were analyzed using Digital Micrograph software.

## Conflict of Interest

The authors declare no conflict of interest.

## Supporting information

Supplementary Material

## Data Availability

The data that support the findings of this study are available from the corresponding author upon reasonable request.
