# Supplementary material for: Layered Ferroelectric NbOI2 Flakes Toward In‐Plane Anisotropic Self‐Powered Sensing
Source: Small Sci. 2023 Nov 29;4(2):2300125. doi: 10.1002/smsc.202300125 (PMC11934979; doi:10.1002/smsc.202300125)
Supplement: Supplementary file 1 — Supplementary Material [file SMSC-4-2300125-s001.pdf]

Supporting information

Layered Ferroelectric $NbOI_2$ Flakes Toward In-Plane Anisotropic Self-Powered Sensing

Xuzhou Sun[a, b], *Yingjie Wan*[a, b], *Yuqiang Fang*[a]*, *Fuqiang Huang*[a, c, d]*

[a] *State Key Laboratory of High Performance Ceramics and Superfine Microstructure, Shanghai Institute of Ceramics, Chinese Academy of Sciences, Shanghai 200050, China*

[b] *Center of Materials Science and Optoelectronics Engineering, University of Chinese Academy of Sciences, Beijing 100049, China*

[c] *Beijing National Laboratory for Molecular Sciences and State, Key Laboratory of Rare Earth Materials Chemistry and Applications, College of Chemistry and Molecular Engineering, Peking University, Beijing 100871, China*

[d] *School of Materials Science and Engineering, Shanghai Jiao Tong University, 800 Dongchuan Road, Shanghai, 200240, China*

* *Corresponding author.*

E-mail: fangyuqiang@mail.sic.ac.cn; huangfq@mail.sic.ac.cn

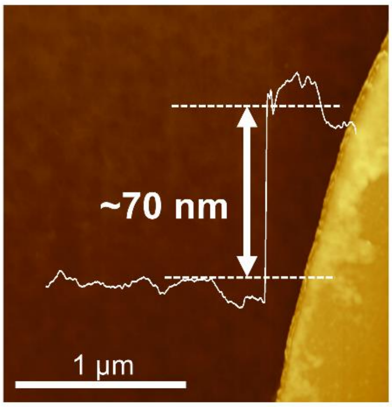

Figure S1. AFM image of $NbOI_2$ in a four-electrode PENG.
This is a typical $NbOI_2$ sample thickness for mechanical stripping.

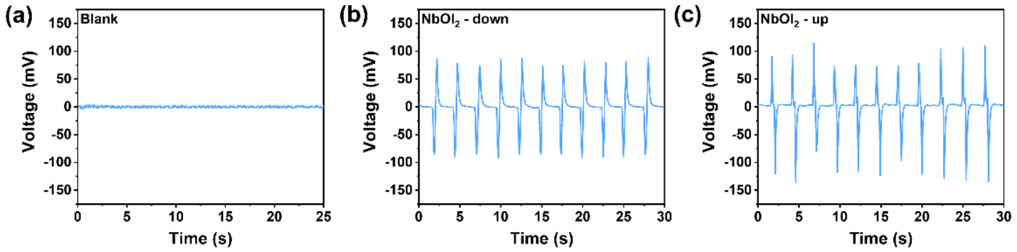

Figure S2. a) Response voltages from bare PET substrate without covering exfoliated $NbOI_2$ flake under periodic strain. Output voltage generated by the $NbOI_2$ device under the b) tensile strain and c) compressive strain.

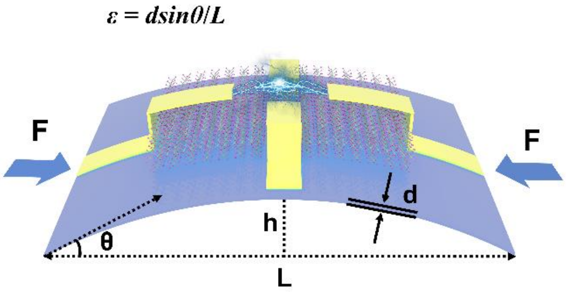

Figure S3 Schematic diagram of the calculation of strain in $NbOI_2$-based PENG. The strain applied to the device can be defined by the formula $\varepsilon = dsin\theta/L$, where, ε is the strain, d is the thickness of the PET, h is the distance from the bending center of the device to the horizontal line, L is the length from the one end of the substrate to the other, and θ is the angle between the tangent line and the horizontal line.

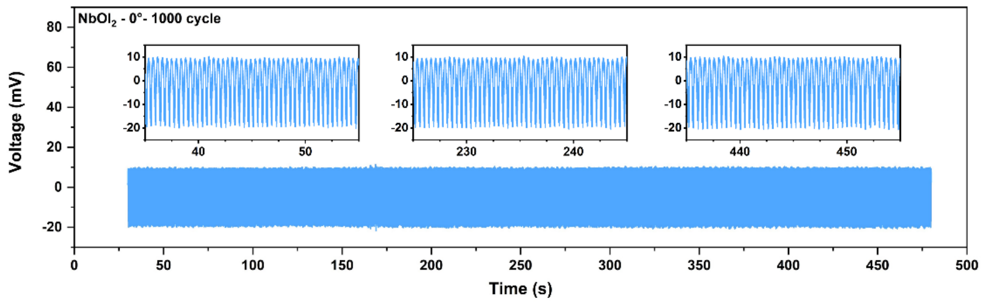

Figure S4. 1000 cycles of V_{OC} under 1.1% strain in 0° direction.

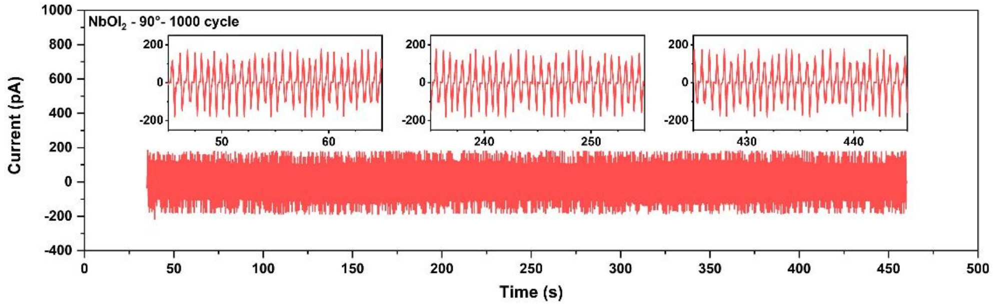

Figure S5. 1000 cycles of I_{SC} under 1.1% strain in 90° direction.

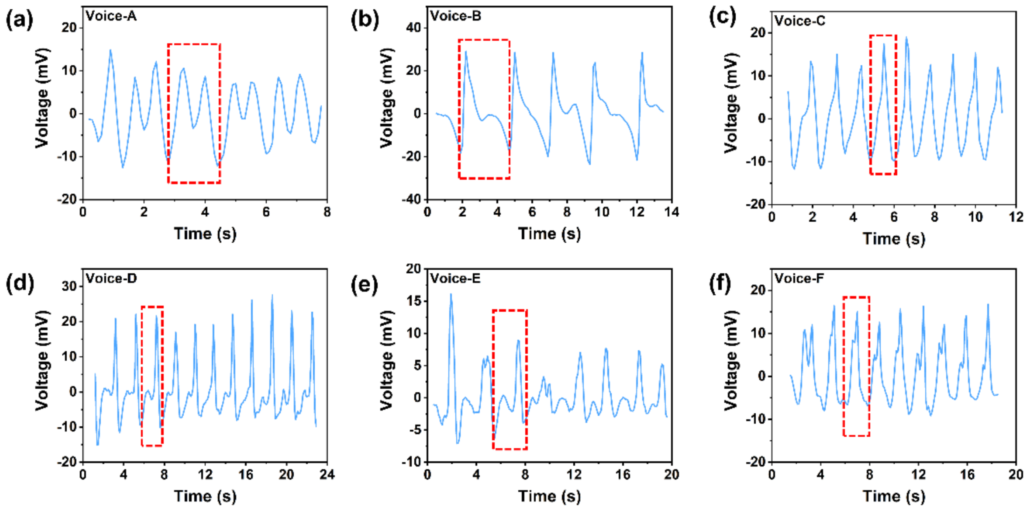

Figure S6. Identify the different letters using PENG, a) to f) are the letters "a" to "f" respectively. The measurement output waveform has high repeatability. (The signal in the dotted box represents a letter sound).
